# Analysis of *TGFBI* gene mutations in Chinese patients with corneal dystrophies and review of the literature

**Published:** 2010-06-30

**Authors:** Juhua Yang, Xiaoli Han, Dinggou Huang, Lin Yu, Yihua Zhu, Yi Tong, Binliang Zhu, Chuanbao Li, Mingshe Weng, Xu Ma

**Affiliations:** 1 Biomedical Engineering Center, Fujian Medical University, Fuzhou, Fujian, China; 2 Department of Genetics, National Research Institute for Family Planning, Peking Union Medical College, Beijing, China; 3 Department of Ophthalmology, The First Affiliated Hospital of Fujian Medical University, Fuzhou, Fujian, China; 4 Fuzhou Southeast Eye Hospital, Fuzhou, Fujian, China; 5 Department of Ophthalmology, The First Hospital of Xi'an, Xi’an, Shanxi, China; 6 Department of Ophthalmology, Affiliated Hospital of Jining Medical college, Jining, Shandong,China; 7 Department of Ophthalmology, Jianou City Hospital, Jianou, Fujian, China

## Abstract

**Purpose:**

To analyze human transforming growth factor b-induced (*TGFBI*) gene mutations in Chinese patients with corneal dystrophies (CDs).

**Methods:**

Twenty-one families with corneal dystrophies were subjected to phenotypic and genotypic characterization. The corneal phenotypes of patients were documented by slit lamp photography. Mutation screening of the coding regions of *TGFBI* was performed by direct sequencing. An additional 43 families and 3 sporadic patients with *TGFBI* dystrophies from China reported in the literature were reviewed.

**Results:**

Five mutations of *TGFBI* were identified in 21 families with CDs, including one novel small deletion mutation, c.△1838–1849 (p.Δ613–616VAEP), responsible for one variant lattice CD (LCD) family and 4 known mutations, R555W mutation for 10 granular cornea dystrophy type I (GCD1) families, R124H for 5 GCD type II (GCD2), R124C for 4 LCD1, and H626R for one variant LCD. In a cohort of Chinese patients (n=355) with *TGFBI* dystrophies from 64 families and 3 sporadic cases, 19 distinct mutations were found in several different CD subtypes. The 3 most common phenotypes were ranked as follows: GCD1, GCD2, and LCD1. Mutation hot spots at R124 and R555 occurred in >80% of these families.

**Conclusions:**

Our findings extend the mutational spectrum of *TFGBI*, and this is also the first extensively delineated *TGFBI* mutation profile associated with the various corneal dystrophies in the Chinese population.

## Introduction

Corneal dystrophies (CDs) are characterized by the occurrence of bilateral progressive opacities of the cornea that often arise from the deposition of insoluble material in the corneal stroma and lead to visual impairment. Since 1997, multiple phenotypes have been identified that are related to allelic mutations of the human transforming growth factor b-induced (*TGFBI*) gene (OMIM 601692) on human chromosome 5q31 [1]. This gene, originally named beta-ig-h3 (*BIGH3*), comprises 17 exons coding for a unique protein of 683-amino acids, which is produced by both mesenchymal and epithelial cells and denoted as keratoepithelin [1]. At present, more than 50 distinct disease-causing mutations have been identified in *TFGBI* that are associated with different corneal dystrophies, including granular cornea dystrophy (GCD) type I (GCD1; OMIM 121900), GCD type II (GCD2; OMIM 607541), lattice corneal dystrophy type I(LCD1; OMIM 122200), variant LCD, Thiel-Behnke corneal dystrophy (CDTB; OMIM 602082), Reis-Buckler corneal dystrophy (CDRB; OMIM 608470), and epithelial basement membrane corneal dystrophy (EBMD; OMIM 121820). In the present study, we designated all of the cornea dystrophies associated with *TGFBI* mutations as *TGFBI* dystrophies.

Of these mutations, two mutational hot spots have been identified corresponding to R124 and R555 of the TGFBI protein as being the most frequent sites of mutation in various populations [2-7]. Despite *TGFBI* dystrophies represent a clinically heterogeneous group of disorders, a strong correlation between specific mutations at these two positions and the observed phenotypes has been observed: GCD1/R555W, CDTB/R555Q, LCD1/R124C, GCD2/R124H, and CDRB/R124L. These above CDs were also classified as the classic form of *TGFBI* dystrophies [8].

The *TGFBI* mutation spectrum and their clinical consequences have been investigated in patients with CDs in different ethnic populations [2-7]. However, the spectrum of *TGFBI* mutations and the correlation between genotype and phenotype in the Chinese population has not been studied extensively. In this study, we report our findings on 21 new CD families with *TGFBI* mutations, and an additional 43 families and 3 sporadic patients with *TGFBI* dystrophies in a Chinese population collected from the literature [9-31], and delineate extensively the *TGFBI* mutation profile associated with the various corneal dystrophies in China.

## Methods

### Patients and subjects

**Figure 1:**
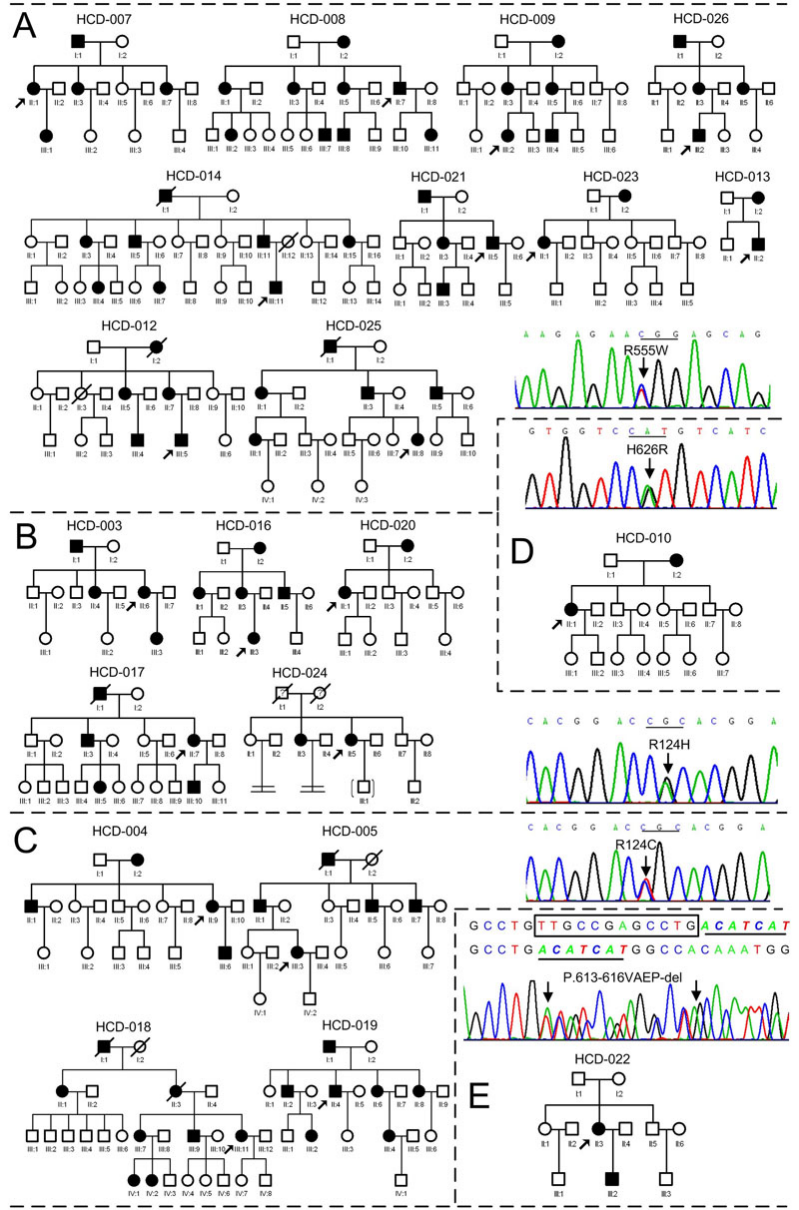
Pedigrees of 21 Chinese families with corneal dystrophies and sequence chromatograms of their corresponding mutations *TGFBI*. GCD 1/R555W mutation (**A**), GCD2/R124H mutation (**B**), LCD1/R124C mutation (**C**), variant LCD/H626R mutation (**D**), and Variant LCD/Δ613–616VAEP mutation (**E**). Squares and circles symbolize males and females, respectively. The open and closed symbols indicate unaffected and affected individuals, respectively. The proband is marked with an arrow.

**Figure 2:**
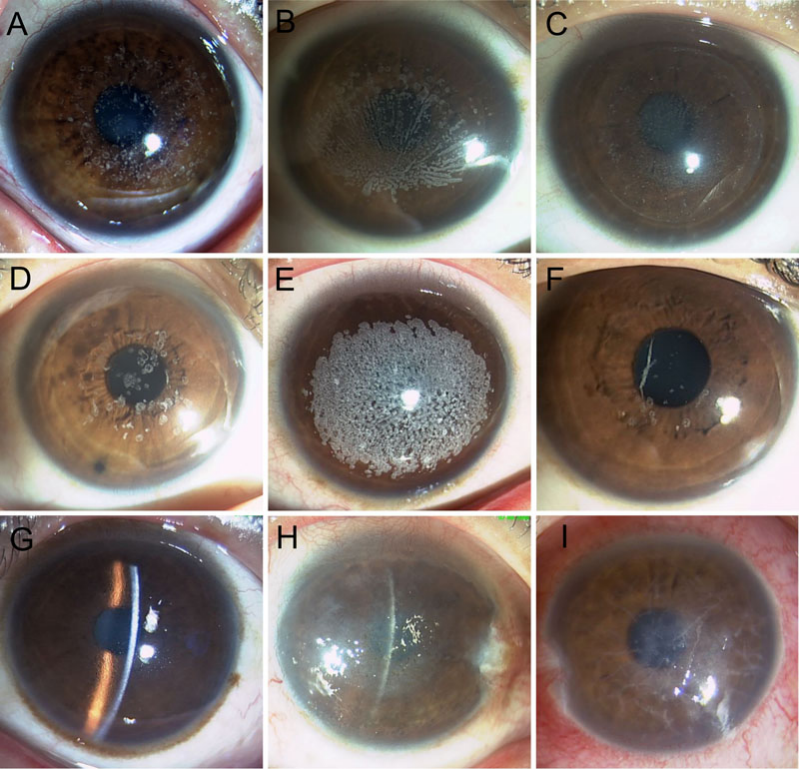
Representative corneal phenotypes as shown by slit lamp examination. The proband of GCD1 family HCD-007 with R555W mutation had crumb-shaped and round white opacities (**A**). The proband of GCD1 family HCD-023 with R555W mutation showed confluent gray white opacities in a fan-like distribution in the central cornea (**B**) and recurrence corneal deposits after penetrating keratoplasty (**C**). The proband of GCD2 family HCD-016 with R124H mutation showed that the corneal opacities resemble rings, disks or snowflakes (**D**). The patient with the severe form of corneal dystrophy had confluent round white opacities in a coralloid shape in the superficial stromal layer (**E**), whereas his brother had fewer round opacities and thick spicular opacities (**F**) in a consanguineous marriage Chinese family clinically diagnosed as GCD2. The proband of LCD1 family HCD-005 with R124C mutation showed numerous fine, branching refractile lattice lines in subepithelial and stromal layers of the cornea (**G**). The proband of LCD family HCD-010 with H626R mutation showed recurrent diffuse stromal haze (**H**). The proband of LCD family HCD-022 with Δ613–616VAEP mutation showed thick, stellate lattice lines with intervening opacities and extending more to the periphery (**I**).

As part of a genetic screening program for genetic eye disorders, we collected materials from 26 families diagnosed with corneal dystrophies from China. A slit-lamp examination was performed for all participating individuals to determine if they were affected or unaffected with corneal dystrophies and to determine the disease phenotype. Of these families, 21 were further classified into 3 diagnostic categories: GCD1 in 10 families, LCD in 6 families, and GCD2 in 5 families. The pedigrees of these 21 families are shown in Figure 1, and the slit-lamp photographs of the representative patients with CDs are shown in Figure 2. After informed consent conforming to the tenets of the Declaration of Helsinki and following the guidance of sample collection of National Infrastructure Program of Chinese Genetic Resources (NICGR), 112 blood samples (58 samples from patients and 54 from normal members of families) from the 21 families and other 50 samples from ethnically matched control individuals were obtained before the study.

### Genetic analysis

Total genomic DNA was extracted from whole blood using the Wizard Genomic DNA Purification Kit (Promega, Beijing, China) according to the manufacturer’s instructions. Amplification of the coding regions of *TGFBI* from genomic DNA was performed with the primers as described previously [1,2,22]. The sequences of the primers are listed in Table 1. Each PCR reaction was carried out in a 50 µl reaction mixture containing 100-200 ng of genomic DNA, 0.25 µl of TaKaRa Ex TaqTM (5U/µl; TaKaRa Biotechnology Co., Ltd., Dalian, China), 4.0 µl of dNTPs (2.5 mM each), 1.0 µl of each primer (10 pmol), 5 µl of 10× Ex Taq Buffer (Mg2+ plus). PCR conditions were as follows: 94 °C for 3 min; 30 cycles of 94 °C for 30 s, 52 °C or 55 °C or 58 °C for 30 s, 72 °C for 1 min; and further extension step at 72 °C for 5 min. PCR products were purified and sequenced on an ABI 3730XL Automated Sequencer (PE Biosystems, Foster City, CA), using the same PCR forward primers.

**Table 1:** Summary of the primers used for the amplification of the 17 exons of the human *TGFBI* gene.

Exon	Primersequnce(5’→3')	Annealing temperature (°C)	Amplicon length (bp)
1	F: GCGCTCTCACTTCCCTGGAG	52	252
	R: GACTACCTGACCTTCCGCAG		
2	F: GGTGGACGTGCTGATCATCT	58	194
	R: AGCCAGCGTGCATACAGCTT		
3	F: ACCTGTGAGGAACAGTGAAG	58	200
	R: GCCTTTTATGTGGGTACTCC		
4	F: CCCCAGAGGCCATCCCTCCT	58	225
	R: CCGGGCAGACGGAGGTCATC		
5	F: TAAACACAGAGTCTGCAGCC	58	260
	R: TTCATTATGCACCAAGGGCC		
6	F: TGTGTTGACTGCTCATCCTT	58	317
	R: CATTCAGGGGAACCTGCTCT		
7	F: TTCAGGGAGCACTCCATCTT	55	224
	R: ATCTAGCTGCACAAATGAGG		
8	F: CTTGACCTGAGTCTGTTTGG	58	324
	R: GAAGTCGCCCAAAGATCTCT		
9	F: ACTTTTGAACCCACTTTCTC	58	200
	R: CAATCTAACAGGGATGCCTT		
10	F: TCTGGACCTAACCATCACCC	58	206
	R: CAGGAGCATGATTTAGGACC		
11	F: CTCGTGGAAGTATAACCAGT	58	223
	R: TGGGCAGAAGCTCCACCCGG		
12	F: CATTCCAGTGGCCTGGACTCTACTATC	58	318
	R: GGGGCCCTGAGGGATCACTACTT		
13	F: GGGATTAACTCTATCTCCTT	58	249
	R: TGTGTATAATTCCATCCTGG		
14	F: CTGTTCAGTAAACACTTGCT	58	262
	R: CTCTCCACCAACTGCCACAT		
15	F: CACTCTGGTCAAACCTGCCT	58	147
	R: AGGCTAGGCGCAAACCTAGC		
16	F: CAGTTGCAGGTATAACTTTC	58	120
	R: TAAACAGGTCTGCAATGACT		
17	F: GGGAGATCTGCACCTATTTG	58	113
	R: TGGTGCATTCCTCCTGTAGT		

### Publications selection

To evaluate the incidence of *TFGBI* mutations in Chinese patients with *TGFBI* dystrophies, we also reviewed published articles obtained by an electronic search on PubMed/MEDLINE, VIP Information, CNKI, WANGFANG, data and Google using the keywords “corneal dystrophy” and “TGFBI” or “BIGH3.” Articles were limited to full papers in the English or Chinese language literature, and the search ended in February, 2010. When the same author reported results obtained on a complete or partial similar patient population in several publications, only the most recent or the most complete report was included in the analysis to avoid overlap. According to these criteria, 23 eligible trials published between 2002 and 2010 were selected [9-31]. The articles reported mutations of the *TGFBI/BIGH3* gene associated with corneal dystrophies in the Chinese population. All of these *TGFBI* dystrophies were reclassified as classic or variant form according to Aldave and Sonmez’s [8] criterion.

## Results

### Mutational analysis

We screened the coding regions of *TGFBI* using PCR-based ampliﬁcation followed by direct sequencing. Mutations were identified in all patients from the 21 unrelated Chinese families (Figure 1). Five distinct mutations of *TGFBI* were identified, including one novel small deletion mutation, c.Δ1838–1849 (p.Δ613–616VAEP), in exon 14 responsible for the HCD-022 family with variant LCD (Figure 1E and Figure 2I). This mutation was found in the proband and her son, but not in her parents and 50 normal controls. The other four known mutations were R555W mutation for 10 GCD1 families (Figure 1A and Figure 2A-C), R124H for 5 GCD2 families (Figure 1B and Figure 2D), R124C for 4 LCD1 families (Figure 1C and Figure 2G) and H626R for one variant LCD family (Figure 1D and Figure 2H).

### Profile of *TGFBI* dystrophies

To delineate the *TGFBI* mutation profile associated with the various corneal dystrophies in the Chinese population, an additional 43 families and 3 sporadic cases with *TGFBI* dystrophies collected from the literature were also included in the investigation. Thus, a total of 355 patients from 64 families and 3 sporadic patients were included in the statistical analysis. We reclassified these *TGFBI* dystrophies as classic or variant form, with GCD1/R555W, GCD2/R124H, LCD1/R124C, CDRB/R124L, and CDTB/R555Q as classic forms and the others as corresponding variants [8].

In this cohort of Chinese patients, 19 distinct *TGFBI* mutations were identified in several different CD subtypes (Table 2). The most common phenotypes were the GCD1 associated with the R555W mutation (26.6%, 17/64 families), GCD2 with the R124H mutation (26.6%,17/64families), and LCD1 with the R124C mutation (18.8%,12/64 families). The mutational hot spots at positions R124 and R555 occurred in 53.1% (34/64) and 28.1% (18/64) of the families, respectively. Our observation also showed that the phenotypes correlated strongly with specific mutations in *TGFBI*, including that between GCD1 and R555W, GCD2 and R124H, and LCD1 and R124C. The exception is two CDTB families associated with the R124C mutation [26]. In addition, more than half (11/19) of these mutations were observed in Chinese patients with variant LCDs (Table 2).

**Table 2:** *TGFBI* mutations and phenotypes of patients in 61 families and 3 sporadic cases from Chinese origin.

**Corneal phenotype**	**Mutation**	**Exon**	**Number of families**	**Number of patients (Male/Female)**	**Reference**
GCD1	R555W	12	17	92 (37/65)	[10-12,17,18,23,28], This study
	A546D	12	1	10 (6/4)	[23]
GCD2	R124H	4	17	65 (32/33)	[10-12,17,24,25], This study
	R124H^+^c.Δ307–308CT	4		1	[9]
LCD1	R124C	4	12	89 (43/46)	[15,16,21,25,31], This study
Variant LCD	H626R	14	3	14 (7/7)	[15,25], This study
	V505D	11	1	10 (4/6)	[14]
	T538P	12	1	3 (1/2)	[16]
	V625D	14	1	2 (1/1)	[20]
	Δ613–616VAEP	14	1	2 (1/1)	This study
	I522N	12	1	4 (1/3)	[29]
	P501T	11		1	[16]
	R514P+F515L	11	1	5 (4/1)	[31]
	A546T	12		1	[30]
	H572R	13	1	3 (2/1)	[31]
CDRB	R124L	4	3	14 (3/11)	[10,13]
	G623D	14	1	7 (3/4)	[22,27]
CDTB	R555Q	12	1	14 (6/8)	[17,19]
	R124C	4	2	18 (9/9)	[26]
Total	19		64	355	

## Discussion

We searched for mutations of *TGFBI* in 21 unrelated Chinese families affected with GCDs or LCDs. Five distinct mutations of *TGFBI* were identified in all of the families (Figure 1), and there have been perfect correlations between GCD1 and R555W, between GCD2 (ACD) and R124H, and between LCD1 and R124C. Among of these mutations, one novel 12 bp deletion mutation, c.Δ1838–1849 (p.Δ613–616VAEP), in exon 14 was found in the HCD-022 family (Figure 1E) with variant LCD (Figure 2I). Compare with the five small deletion mutations (1~6 bp deleted) in *TGFBI* reported previously by other groups [2,4,9,32,33], this mutation was the longest deletion mutation in *TGFBI*. It was also identified as a spontaneous inheritable *TGFBI* mutation because only the proband and her affected son carried the mutation, and not her parents. Spontaneous mutations in this gene are rare, but have been reported. Tanhehco et al. [34] reported a spontaneous R124L mutation in *TGFBI* in 2 patients with CDRB, and Zhao et al. [35] reported a spontaneous R555Q mutation in *TGFBI* in two unrelated families with Bowman's layer corneal dystrophies (CDTB and CDRB). Our finding not only extends the mutational spectrum of *TFGBI*, but also has historic value as a possible explanation for the presence of this autosomal dominant disorder in the population.

Through extensive investigation of the clinical and genetic findings in *TGFBI* dystrophies in the Chinese population, we observed that GCD1 in association with the R555W mutation (17 families) and GCD2 in association with the R124H mutation (17 families) accounted for more than half (53.1%, 34/64) of the families and were the most prevalent type of this disease. Our observations also confirm the presence of mutation hot spots at positions R124 and R555, which occur in more than 80% (52/64) of the families with *TGFBI* dystrophies in China. There were strong genotype-phenotype correlations between R555W and GCD1, between R124H and GCD2, and between R124C and LCD1 in our collection of 64 families, which is consistent with previous reports in patients from other ethnic origins [2-7]. The exception is 2 Chinese typical CDTB families associated with the R124C mutation [26]. Although these two mutations have been identified as major causes of LCDs or GCDs in different ethnic groups [2-7], population-specific variations in the prevalence of different mutations in *TGFBI* have been documented. The classic form of GCD1 with the R555W mutation and LCD1 with the R124C mutation are more common in different ethnic groups in Europe, the United States, and India [2,4,6,7], but in other populations, such as in Japan and Korea, GCD1 is found much less frequently than GCD2 associated with the R124H mutation [3,5], and H626R may be more frequent than R124C in Vietnamese patients with LCDs [36].

The second common type of TGFBI dystrophies is the LCDs, which accounted for more than one third of all of the families (34.4%, 22/64) in the Chinese population. Of these LCDs families, the major subtype is the classic form of LCD (LCD1) associated with the R124C mutation (54.5%,12/22). The other different forms of lattice dystrophy were reclassified as variant LCDs, which were the most mutationally heterogeneous and accounted for half of the mutations identified. In comparison to these common types, CDRB and CDTB were rare (about 5% of each subtype) in the Chinese population.

Most of the *TGFBI* mutations associated with corneal dystrophies are heterozygous. However, homozygous mutations are also found in some patients with severe form of *TGFBI* dystrophies [37,38]. Only two patients bearing homozygous R124H mutations from a consanguineous marriage family with GCD2 have been reported in China [24]. This disease is also described as a semidominant condition in which the heterozygous mutant is less severely affected than the homozygote. We also found a patient with severe form of corneal dystrophy in a consanguineous marriage family clinically diagnosed as GCD2. He had confluent round opacities in a coralloid shape in both of his eyes (Figure 2E), which is similar in phenotype to the patients with GCD2 bearing a homozygous R124H mutation reported previously [38]. His parents and brother had fewer round opacities and spicular opacities (Figure 2F). Unfortunately, we could not obtain blood samples for gene examination.

In addition to allelic heterogeneity, *TGFBI* dystrophies also exhibited extensive intrafamily and interfamily variations in clinical phenotypes, even when the same single point mutation in *TGFBI* is involved. Perhaps the most extreme example of the phenotypic variability is unaffected carriers who had the disease-causing mutations in *TGFBI*. In our collection of 64 Chinese families, 11 these carriers from 4 families were reported [21,23,24]. Of these cases, 2 unaffected relatives bearing the A546D mutation from a GCD1 family [23] and 2 from 2 LCD1 families carrying the R124C mutation [21] were too young to rule out inheritance of these two diseases by clinical examination. However, 7 individuals carrying a heterogeneous R124H mutation showed no clinical manifestations in a reduced penetrance GCD2 Chinese family, and an age-related factor was excluded in the contribution to these carriers [24]. All of this clinical evidence suggests that other factors such as age, environmental factors, and modifier genes may influence the expressivity and the penetrance of this disease, but further studies are needed for confirmation.

In summary, this study reported our findings in 21 new CD families with *TGFBI* mutations, and delineated extensively the *TGFBI* mutation profile associated with the various corneal dystrophies in the Chinese population: both GCD1 associated with the R555W mutation and GCD2 with the R124H mutation are the most common forms of this disease. We also confirmed the presence of mutational hotspots at positions R124 and R555, and a strong correlation between these two mutations and their phenotypes.
